# Tuning the Superhydrophobic Properties of Hierarchical Nano-microstructural Silica Biomorph Arrays Grown at Triphasic Interfaces

**DOI:** 10.1038/s41598-020-61530-0

**Published:** 2020-03-12

**Authors:** Xu-Fu Xiang, Peng-Jie Li, Bi-Feng Liu

**Affiliations:** The Key Laboratory for Biomedical Photonics of MOE at Wuhan National Laboratory for Optoelectronics-Hubei Bioinformatics & Molecular Imaging Key Laboratory, Systems Biology Theme, Department of Biomedical Engineering, College of Life Science and Technology, Huazhong University of Science and Technology, Wuhan, 430074 China

**Keywords:** Materials science, Nanoscience and technology

## Abstract

The three-dimensional hierarchical morphology of surfaces greatly affects the wettability, absorption and microfabrication properties of their hybrid materials, however few scalable methods exist that controls simultaneously complex geometric shape and spatial scattered location and their physical properties tuned. Consequently, this report describes a synthetic strategy that enables the position of well-ordered biomorph nano-microstructures on hydrophobic surfaces to be precisely controlled. The hierarchical architecture can be accurately positioned on polydimethylsiloxane (PDMS) surfaces in an unprecedented level by leveraging a solid/liquid/gas triphase dynamic reaction diffusion system strategy. The effect of salt concentrations, pH, CO_2_ levels, temperature and substrate patterning on this self-assembly process has been investigated, enabling protocols to be devised that enables the hydrophobic properties of the hierarchically assembled multiscale microstructures to be tuned as required. This combined top-down/bottom-up approach can be used to produce composites with outstanding hydrophobicity properties, affording superhydrophobic materials that are capable of retaining water droplets on their surfaces, even when the material is inverted by 180°, with a wide range of potential applications in oil/water separation technology and for selective cell recognition in biological systems.

## Introduction

Self-assembly processes present in nature have inspired scientists to develop the artificial supramolecular self-assembly systems for the synthesis of complex and hierarchical structures that allow functional materials that exhibit useful physical/chemical/structural properties to be produced at scales ranging from nanometers to millimeters^1^. In particular, the well-ordered patterns and aligned hierarchical morphologies produced by synthetic inorganic materials (e.g., silica-based systems) can impart unique anisotropies, large surface areas and enhanced charge transport properties to materials for applications in opto-electronic devices^2–4^, catalysis^5^, mechanics^6,7^, superwettable materials^8,9^ and molecular diagnostics^10^. However, the development of efficient self-assembly techniques that enable directed mesoscale complex construction using low-cost scalable protocols for the production of a wide range of materials is still urgently required.

Self-assembly processes that occur during biomineralization at ambient temperature afford highly functional materials with hierarchical architectures and stunning beauty that have prompted the development of rationally designed silicon-based materials. For example, silica-carbonate biomorphs comprised of encapsulated crystalline carbonate and amorphous silica have been synthesized by diffusing CO_2_ into alkaline solutions of sodium silicate (affording CO_3_^2−^ and H^+^ ions) that results in the coprecipitation of SrCO_3_ and SiO_2_^11–17^. This simple approach involves mixing of cheap ingredients (Na_2_SiO_3_ and Sr/BaCl_2_) to create silicon composites with unprecedented complex morphologies (vases, corals, flowers, worms and plates) that are generated through randomly dispersed growth processes^12,18^. The key factor in these bioinspired mineralization systems is the presence of front reaction-diffusion processes that create the dynamic environment required for production of these precisely sculpted arbitrary architectures^19–24^. These processes serve to create an artificial microenvironment that creates exactly the right chemical and thermodynamic conditions required for spatial control of the microstructures that are produced^25^. Initial reports into generating silica biomorph systems in dilute silica sols or silica gels involved immersion of a substrate into a solution containing reactive silicon precursors which resulted in the random generation of microstructures with no positional control^12,13,16,17^. More recently, a bubble mediated substrate water/air interface strategy has been developed to induce crystallization at solid-liquid-gas triphasic contact lines that can be used to control the spatial arrangement and morphology of the self-assembled surfaces on polydimethylsiloxane (PDMS) surfaces^26^. This approach relies on the hydrophobic micropillar-based PDMS surfaces containing air gaps that enable reagents in solution to be effectively transported to their edges where they can then react to form defined microstructures. In these processes, the micropillars of the PDMS act as a solid support that simultaneously interacts with air (containing CO_2_ gas) and the solution (containing reactive silicon reagents) to produce a solid-liquid-gas triphasic reaction that deposits microstructures on the surface of the micropillars. The incorporation of soft lithography techniques into this hydrophobic micropillar fabrication process, can potentially control the location of the self-assembly growth process to afford selectively patterned materials. Three-dimensional microscopic computed tomography (3D-CT) technology has been used to determine the amount of air in contact with the liquid/solid phases, thus enabling the structure of the gas transfer networks present in these systems to be determined. These studies have shown that this gas transfer network is essential, because it sustains the integrity of the liquid phase and facilitates solution based gas diffusion-reaction processes that are required for self-assembly to occur at room temperature. Aebisher and co-workers^27^ have reported other types of reactive solid-liquid droplet-based systems, employing silicon phthalocyanine sensitizing particles to study the role of hydrophobic photosensitizers that generate oxygen gas in droplets for the functionalization of PDMS micropillars. Furthermore, Wang^28^ and Wu^29^ have reported diffusion studies on H_2_ and H_2_S at triphasic interfaces under nonequilibrium conditions that enabled gold and metal sulfide dish-like microstructural arrays to be grown on the top surfaces and edges of PDMS pillars. Therefore, the ability of well-organized hydrophobic micropillar substrates to act as structural templates to precisely locate gas-liquid-solid triphasic interfacial self-assembly reactions has been firmly established.

In this study, we report how self-assembly processes generated through bottom up biomineralization and top-down lithography processes that occur at hydrophobic gas-liquid-solid interfaces can be used to generate multiscale patterned architectures on pillar templates. Optimization of reaction times, reagent concentrations, pillar geometries and temperatures of this triphasic self-assembly process has enabled reproducible tunable protocols to be developed that enable the scalable interfacial growth of uniform SrCO_3_–SiO_2_ microstructural arrays on hydrophobic micropillar substrates. These protocols generate stable CO_2_ concentrations at the reactive triphasic interface of these hydrophobic self-assembly processes, thus enabling the location and hierarchical structures of the microstructures formed on the pillars to be studied and controlled. This new triphasic interface approach represents a novel platform for investigating crystal growth mechanisms for the formation of carbonate-silica biomorphs and is likely to be of use for the production of silicon-based arrays of use in functional devices.

## Experimental Section

Growth of carbonate/silica structures: A PDMS micropillar substrate was positioned vertically in a 100 mL beaker containing 15 ml aqueous solution of Sr/BaCl_2_ and Na_2_SiO_3_ (Sigma Aldrich) using the set-up shown in Table S1. The reaction vessel was then loosely covered with a Petri dish to allow CO_2_ from the atmosphere to diffuse into the system. The pH of the solution was measured using a Mettler Toledo FE20 FiveEasy pH meter and adjusted to between 10 to 13 by adding HCl or NaOH, respectively (see Table S2). After 12 h, the experiment was stopped by carefully adding the samples to pure water which terminated the reaction process. Samples were then placed in acetone for 3 min (to replace water with a low surface tension liquid) and carefully dried using a vacuum freeze-drying machine.

Characterization of functionalized materials: Sample analysis was performed using a VHX-2000C three-dimensional stereomicroscope (3D-CT); a FEI Nova NanoSEM scanning electron microscope (SEM) equipped with a SE2 detector and a four-quadrant electron backscatter detector; and a JEOL JEM-2100F TEM (EM-20014, UHR, 200 kV, Japan) equipped with a F-216 digital camera (TVIPS, Germany), a high angle annular dark field (HAADF) detector and an INCA X-SIGHT EDX system (OXFORD, UK). Thin film X-ray diffraction (Shimadzu 6000 with Cu Kα radiation (λ = 1.5418 Å)) was used to analyze phase compositions of all coatings using a grazing angle of 1°. High resolution transmission electron microscope (HRTEM) imaging and energy dispersion X-Ray spectroscopy (EDS) were employed to further characterize the crystalline structure and chemical composition of the crystalline products.

Determining Superhydrophobic Properties of Materials: A water droplet was placed onto the surface of the self-assembled composite materials using a syringe needle. Pictures of formed droplets were taken using an optical microscope from the side view and the resultant images analyzed using “Image J Software”, that calculated contact angles for materials with the different number of arms and gaps.

## Results and Discussion

**Figure 1:**
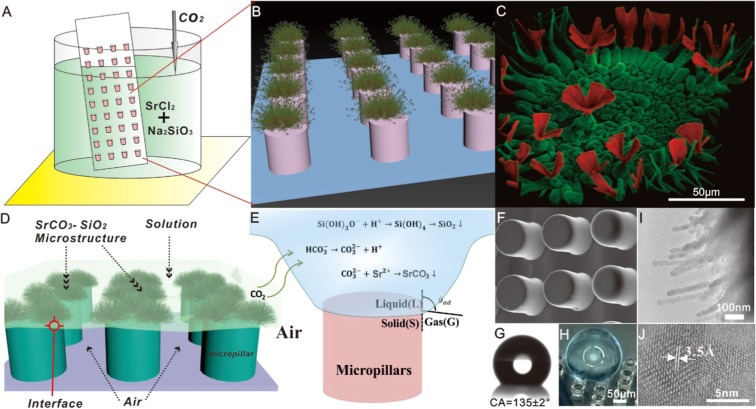
Schematic and SEM/TEM images of fabricated silica biomorphs: (**A**) Micropillars vertically submerged in solution with an initial pH of 11.8. (**B**,**D**,**E**) Schematic illustration of hierarchical architectural formation occurring at the interface between the micropillar arrays and the air pocket. (**C**,**F**) SEM images of SrCO_3_-SiO_2_ structures formed at the top of the micropillar edges. (**G**) Photographic image of a 1.5μm water droplet on a surface with a contact angle (CA) of 135 ± 2°. (**H**) 3D-CT view of a 400μm diameter droplet on a hydrophobic pillar-structured substrate. (**I**,**J**) TEM/HRTEM image of the SrCO_3_ nanostructure.

PDMS micropillar structure was fabricated using conventional photolithographic processes to produce patterned surfaces with typical dimensions of 100 μm width, 50 μm gap, and 100μm height (Fig. 1F), with all substrates being dried thoroughly in a vacuum before being used in self-assembly experiments. The rough surfaces of the exposed PDMS surfaces exhibited hydrophobic properties with a water contact angle of 135 ± 2° (Fig. 1G–H) and were found to contact SrCl_2_–Na_2_SiO_3_ solution (via air pockets) in a CO_2_ atmosphere at room temperature (Fig. 1E). Fabrication of the SrCO_3_–SiO_2_ architectural arrays was achieved by vertically immersing the PDMS substrate in a beaker containing an aqueous solution of SrCl_2_ (19.1 mM) and Na_2_SiO_3_ (8.2 mM) at pH of 11.8. The beaker was then fitted with a loose cover that allowed gas diffusion from the atmosphere to the surface of the pillars (Fig. 1A,D) and then placed in an incubator whose atmosphere and temperature could be regulated. The incubator was then pressurized with CO_2_ and left for several hours to allow the hierarchical nano-microstructures to grow on the surfaces of the pillars of the PDMS substrate (Fig. 1C). Alcohol was then added to the solution to decrease its surface tension, which enabled the fragile PDMS chip microstructures to be isolated without damage, which was then freeze-dried under vacuum. The analysis revealed that triphasic reactions had resulted in regular SrCO_3_-SiO_2_ hierarchical structures being deposited at the top and edge regions of the PDMS pillars, with no deposition having occurred on their bottom regions. Protruding branches were present inside the pillar gaps located at the liquid-gas interface, as observed previously when bulk solution air-water interface condition was employed. Analysis by scanning electron microscopy (SEM) revealed that the carbonate-silica biomorphs with microflower and micrograss morphologies were present on the surface of the PDMS pillars (see Fig. 1C). Energy dispersive spectrometric (EDS) analysis confirmed that the microstructures were comprised of silica (Fig. S8). Magnified TEM images confirmed the presence of protruding arms with relatively large surface areas of 20 nm width and 200-400 nm length (insert in Fig. 1I). The amorphous silica deposits affording a single bright diffraction spot, whilst X-ray diffraction (XRD) patterns clearly revealed CP2 diffraction peaks expected for the body-centered cubic structure of SrCO_3_ (JCPDS no. 14941-1-40-3) (Fig. S2B insert). Therefore, these analytical results confirm that the interface-induced microflower-shaped constructs produced in these self-assembly processes are composed of polycrystalline SrCO_3_ and amorphous silica building blocks.

**Figure 2:**
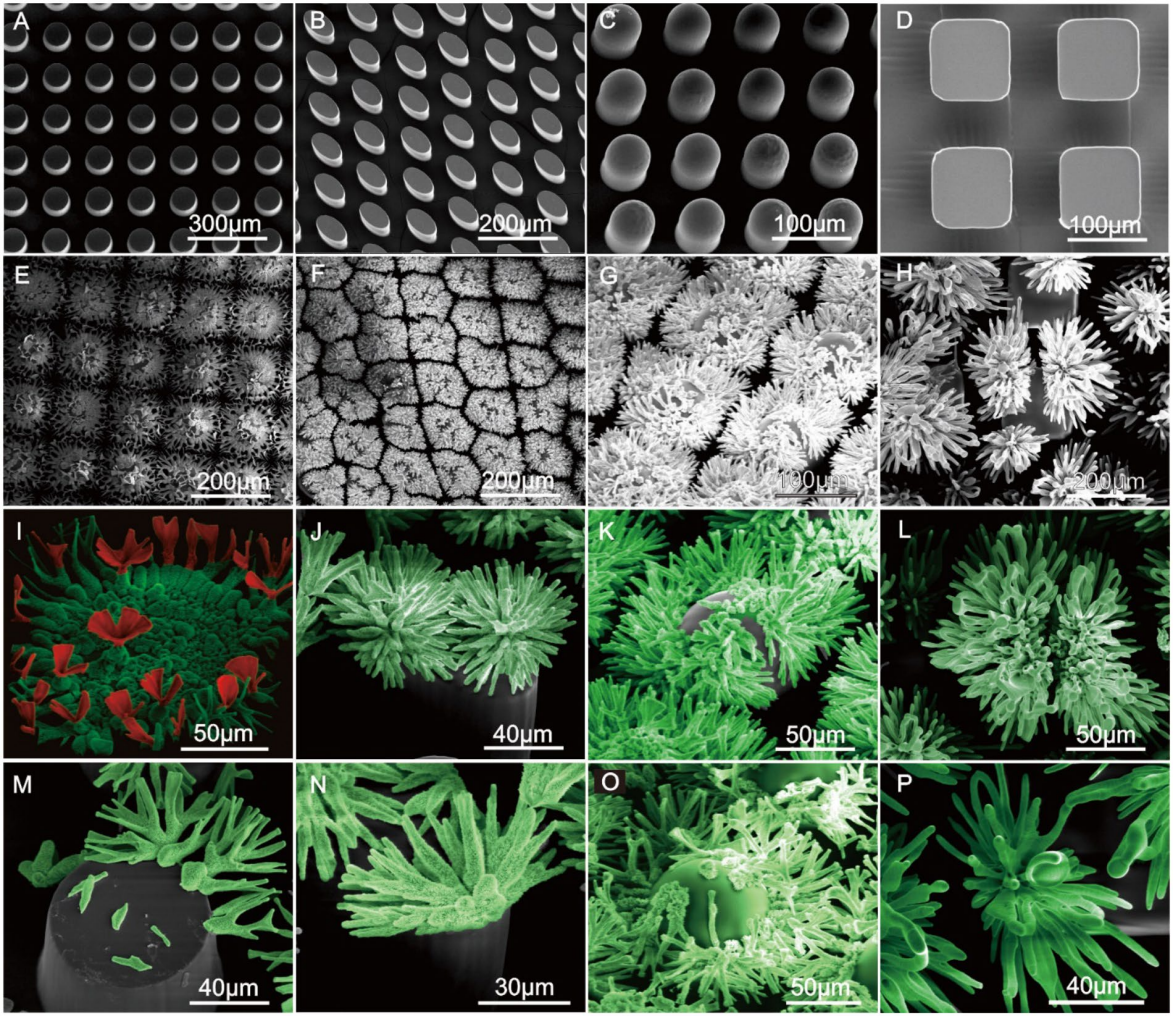
SEM images of the growth morphology of different micropillar patterned silica biomorphs on (i) cylindrical micropillars (**A**,**E**,**I**,**M**); (ii) elliptical micropillars (**B**,**F**,**J**,**N**); (iii) rounded nose micropillars (**C**,**G**,**K**,**O**), (iv) square micropillars in solution (**D**,**H**,**L**,**P**). Biomorphs formed using a solution of BaCl_2_ (19.1 mM) and Na_2_SiO_3_ (8.2 mM) at pH 11.8.

Nucleation and crystallization processes at triphasic interfaces controls the location of self-assembly in these systems, with air-water interfaces unable to support crystal nucleus growth. Nucleation processes generally occur at surface edges with small radii of curvature, which means the self-assembly process only occur at the triphasic solid-liquid-gas interface. The rate of microstructural growth at the top of the pillar (bulk liquid) is slower than that at the edges because the concentration of CO_2_ required for the reaction is less at this more exposed interface (Fig. 2C,G,K,O). The air pockets in the gaps in the pillars provides a link between the triphasic interface and the CO_2_ atmosphere, with the bulk solution containing the reactive silicon precursors contacting each vertical PDMS pillar that is exposed to a differential CO_2_ gradient (Fig. 1D). Therefore, this triphasic architecture clearly has the potential for the production of multilayer surfaces with different shapes and hydrophobicities for the production of tunable silicon based microarrays for applications in chip design (Fig. 2).

Attempts to carry out triphasic interface coprecipitation reactions using patterned pillars with 100 μm gaps proved unsuccessful, because the larger liquid surface area between the two pillars generated a large amount of sediment which resulted in the architecture being disrupted and liquid flowing directly into the gaps to displace the air voids. Similarly, when the micropillars were inclined at an angle (other than 90°) in the solution, then the uncontrolled aggregation of SiO_2_ and SrCO_3_ resulted in the liquid entering the gaps to disrupt the self-assembly process. Consequently, all self-assembly reaction was carried out using vertically aligned PDMS templates to avoid the air-liquid interface from being disrupted and ensure free CO_2_ exchange between the atmosphere and the triphasic reaction sites (Fig. 2 and Movie S1 S2).

The structural growth behaviors of root, stem and petal segments of interface-mediated silica biomorphs were investigated over time (Fig. S2A). Root segment growth proceeded quite slowly, indicating that a relatively low concentration of SrCO_3_ and SiO_2_ were present at the reactive triphasic sites in these systems. Initial nucleation events occur at the pillar top edges that provide high surface energies and tensions to establish effective triphasic interfaces for functionalization of the micropillar surface. After an hour, self-assembles stems were observed with a statistical average length of 5 μm, whose formation occurs because of continuous diffusion of CO_2_ gas through the ambient-connected gas network, which facilitates the solid crystallization process. Over the next five hours, the width of the stem remains constant at 2 μm and its length rising to 40–45 μm as the number of crystals present reached saturation level. Between seven and nine hours, the rate of the self-assembly reaction process decelerated significantly, which is probably due to the pH value at the interface being reduced and the surface wetting properties of the system change. A concomitant reduction in ion concentration levels results in a change in growth behavior to a radial mode that produces agglomerates with plate vase like shapes. To demonstrate that lower ion concentration was responsible for this change in structural morphology, we repeated pillar functionalization experiments using a solution containing half the concentration of the reactive silicon precursors, which resulted in the formation of vase shape assemblies. Notably, once the tops of pillars were uniformly coated by the hierarchical architecture of a definite size, then the wetting properties of the exposed surfaces became hydrophilic, with the pillar gaps still being exposed to CO_2_ gas. Interestingly, PDMS pillars whose surfaces were pretreated with oxygen plasma to produce hydrophilic surfaces produced microstructural assemblies wit rambling structures, similar to those produced in standard solution based self-assembly systems.

The effect of different temperatures on the growth of silica biomorph architectures was also investigated, with increasing temperatures resulting in increased reaction rates due to air bubbles destroying the gas-liquid interface, which results in the liquid fully permeating into the pillar gaps to afford less morphologically defined microstructures. Conversely, when the temperature declines, the growth rate decreases, with no surface based self-assembly processes found to occur at 0 °C. As a result, it is recommended that a temperature range of between 25–45 °C (Fig. S2B) is used in these self-assembly reactions, with temperature variation a potentially useful variable that can be used to modify the size and morphology of the resultant structures.

**Figure 3:**
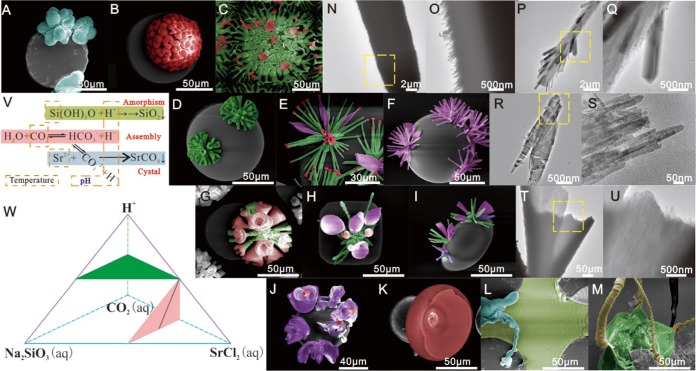
Regulated synthesis of hierarchical complex structures at different solution concentrations and pH. The false-colored SEMs (**A**–**M**) were produced using solution concentrations and pH values described in Table S3. The TEMs (**N**–**U**) represent three basic nanostrutures that appear as coral-like/branch-like, flowerlike and vessellike/wormlike hierarchical assembled microstructures, respectively. These structues include 100–200 nm columnar SrCO_3_ nanocrystals, 20–50 nm width SrCO_3_-SiO_2_ nanowires and 5–10 nm width SrCO_3_-SiO_2_ nanoslices. The schematic chemical reaction (**V**) shows the reactivity relationships (**W**) between the four reactive components (Na_2_SiO_3_, SrCl_2_, CO_2_ and H^+^).

The chemical reaction (see Fig. 3V) underpinning this self-assembly reaction takes place under alkaline conditions (pH 9–13) and relies on changes in speciation of each reactive component being induced by precipitation of another component. As barium carbonate nucleates and crystallizes at the front of a developing aggregate, then the localized decrease in concentration carbonate ion induces dissociation of nearby bicarbonate ions to re-establish the perturbed HCO_3_^−^/CO_3_^2−^ speciation equilibrium. This results in the concomitant release of protons that react near crystallites to dissolve silicate species, such as Si(OH)_3_O^−^. These localized protonation events raise the concentration of silicic acid, resulting in supersaturation of amorphous silica which precipitates out and polymerizes around the carbonate crystallites, thus preventing further growth at that site. Continuous oligomerization reactions remove protons from the precipitation site, which in turn affects local carbonate speciation by increasing the amount of CO_3_^2−^ ions present^11–16,19,21^. This causes an increase in supersaturation of barium carbonate, resulting in the nucleation sequence being repeated and further growth occurring. After an initial stage involving silica-induced fractal branching of a carbonate seed crystal, this dynamic interplay results in emerging nanocrystalline assemblies initially growing as cardioid shaped quasi-2D laminar sheets that are a few microns thick. These sheets can grow along the surfaces of vessel walls, or assemble on top of each other, curling, bending and twisting in the process to produce delicate helical or worm-like morphologies. Based on this knowledge, we investigated whether the morphologies of the silica biomorphs could be controlled by modifying salt concentration and pH levels used in these self-assembly reactions. Inspired by phase diagram theory, there were four factors affecting the chemical reaction (see Fig. 3W); with a green triangle indicating that the concentration of CO_2_ and pH was unchanged for variable amounts of NaSiO_3_ and SrCl_2_; and a pink triangle with a black line indicating that the NaSiO_3_/SrCl_2_ ratio and CO_2_ concentration levels were unchanged for pH various from X to Y (Table S1, S2). These optimized screening reactions identified conditions could be used to produce different types of microstructures on the surfaces of the PDMS pillars like flower, branch, vessel, coral, worm, like and bowl microstructures (Fig. 3A–M) (Table S3). Flower-like microstructures (Fig. 3H) with branch-like architectures were generated in five of the protocols, with the extent of their proliferation through a genetic growth mode controlled by varying growth times. Three types of nanostructure were found to influence the morphology of these systems: columnar 100–200 nm SrCO_3_ nanocrystals; 20–50 nm SrCO_3_-SiO_2_ nanowires; and 5–10 nm SrCO_3_-SiO_2_ nanoslices (Fig. 3N–U). When the amount of glassy SiO_2_ increased, the amount of nanocrystal SrCO_3_ present for growth becomes limited, resulting in different crystalline forms of SrCO_3_ being produced that are then encapsulated in nanoglass SiO_2_. The various branch-like microstructures present were mainly produced on big columnar SrCO_3_ nanocrystals, whilst the leaflike and wormlike shapes ware mostly present on SrCO_3_-SiO_2_ nanoslices, with the rest of the shapes containing all three types of nanostructure. These experiment results fit well with Noorduin’s hypothesis^12^ of structural evolution in this type of self-assembly systems occurring via three distinct growth regimes.

The mechanism of formation of the microcrystalline deposits produced in this system is dependent on the microenvironment (pH, ion concentration, temperature) at the front the oscillatory coprecipitation reaction. Three types of micro-environment interfaces where CO_2_ could potentially be captured may be considered: (1) at a gas-liquid biphasic interface (Fig. S6A); (2) at a solid-liquid biphasic interface (Fig. S6B); and (3) at a gas-liquid-solid triphasic interface (Fig. S6C,D). CO_2_ directly contacted with the solution at a gas-liquid interface can potentially generate an explosive dendritic morphology, with surface tension effects having little effect on their morphology. By contrast, when CO_2_ is consumed more gradually at increased depths in the pillar gaps, then the rough vertical sides of the pillar substrate can induce a variable CO_2_ gradient distribution that negatively affect the uniformity of the self-assembly process. Therefore, accurate adjustment of parameters to account for the effects of this substrate induced CO_2_ concentration gradient are difficult to achieve, and so the presence of the air pocket in the voids in the pillars is required to closely regulate the gas concentration available to the triphasic reaction centers that produce these silica biomorphs system.

**Figure 4:**
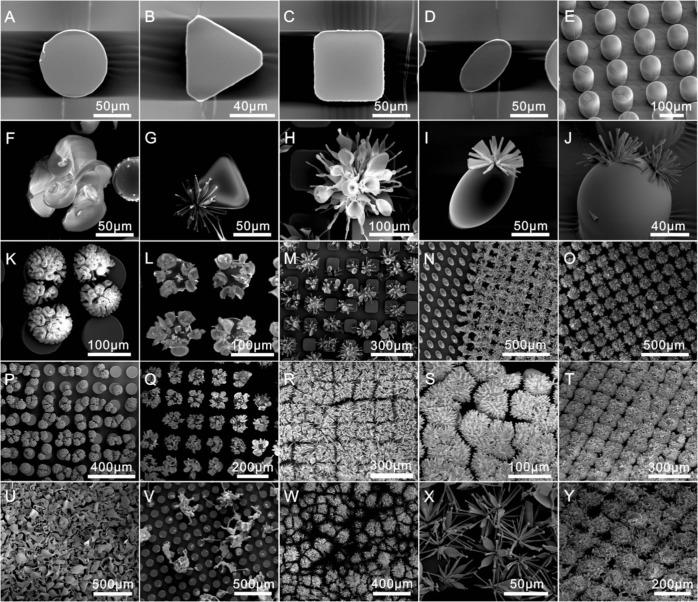
Diversity of structures grown on different geometric micropillars (triphasic interface) under different solution conditions. Images of cylindrical micropillars (**A**,**F**,**K**,**L**,**P**,**Q**,**U**,**V**), triangular micropillars (**B**,**G**), square micropillars (**C**,**H**,**M**,**R**,**W**), elliptical micropillars (**D**,**I**,**N**,**S**,**X**) and rounded nose micropillars (**E**,**J**,**O**,**T**,**Y**) microstructures that were obtained through adjusting solution composition, temperature, growth time and pillar shape.

**Figure 5:**
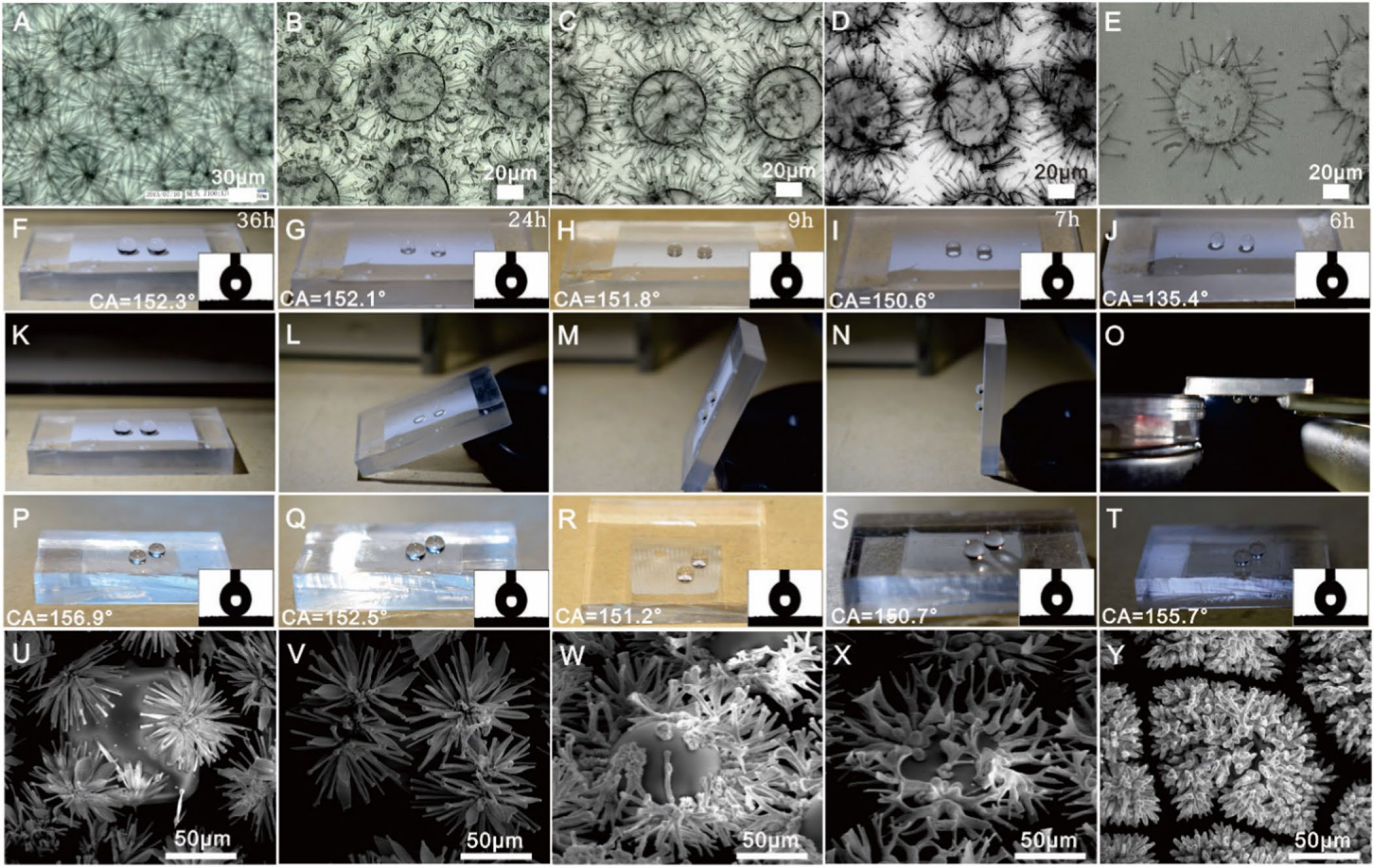
Superhydrophobicity of hierarchical silica biomorph arrays. (i) Time-dependent formation of microflowers affects surface wettability by increasing branch lengths and space distribution densities (**A–E**) to afford hydrophobic and superhydrophobic surfaces (**F–J**). (ii) Images of water droplets adhered to a SrCO_3_-SiO_2_ hierarchical structure rotated through 0°−180° (**K–O**). (iii) Superhydrophobicities of biomorph arrays present on different pillar types (**P–T**) characterized by their different microflower shapes (**U–Y**).

The surface of the template substrate in conventional processes does not influence on the nucleation and growth of crystal formation. However our methodology generates silica biomorphs at a triphasic interface that enables SrCO_3_-SiO_2_ nanostructures with different morphologies to be generated depending on the reaction conditions employed (Fig. 4). Interestingly, the surfaces of most of the complex hierarchical structures produced in this study were shown to exhibit superhydrophobicity (i.e. lotus effect) with a maximum water contact angle of 155.7° being achieved (Fig. 5F–I,P–T). The superhydrophobicity of a solid’s surface is dependent on its hierarchical roughness which in turn determines its surface energy, with the wettability properties of the silicon assemblies grown on the tops of all the different patterned PDMS pillars investigated shown to display superhydrophobic properties (Fig. 5P–Y). The capillary-force-based hierarchical structures of these superhydrophobic silicon coated materials enabled them to retain water droplets when the functionalized material was rotated through different orientations (flat, tilted at 25°, 75° and 90° (Fig. 5K–N, Movie S3). Increasing the size and density of the silica biomorph (Fig. 5A–E) resulted in the superhydrophobicity of the resultant synthetic hierarchical structure being amplified. Indeed, the superhydrophobic surfaces of hierarchical SrCO_3_-SiO_2_ materials were even capable of retaining water droplets when they were inverted through 180° (Fig. 5K–O, Movie S3). As reported, there are four different kinds of contact regime for the eggbeater structure in contact with water W-W, CB-CB, W_M_-CB_m_, CB_M_-W_m_ (W represent Wenzel; CB represents Cassie–Baxter; subscript “M” is for “Macro”, “m” is for “micro”)^30^. As for the mixed CB_M_-Wm state (lower/macro-Cassie–Baxter and upper/micro-Wenzel) with the water droplet pinned on the top of arms and an air layer is trapped by nano-microstructure arms (Fig. 5F–I,P–T). It is noticed that superhydrophobicity is increased with the increment of higher nano-microstructure intensity or the surface roughness, however the roughness too large would make the droplet can’t stand on their surface (Movie S4). Together with the optical microscope images, we believe the presence of a CB_M_-W_m_ regime in the middle and CB-CB regime in the edges when the SiO_2_-SrCO_3_ surface is in contact with water droplet. This CB regime in the edges provides enough liquid air contact area to support the super-hydrophobic effect, and the micro-Wenzel (Wm) state in the middle makes the high CA hysteresis and petal effect (Fig. 5K–N, Movie S3).

The long-term stability of the superhydrophobicity of the obtained surface after humidity, outdoor exposure and UV for various time intervals was also investigated. The relative air humidity was controlled by using the saturated salt solution method according to EN ISO 483. The samples were put into a closed container with different kinds of saturated salt solutions by which the relative humidity in the container could be kept constant. Hydrophobicity transfer characteristics of microstructure arrays are shown in Fig. S7A. It can be seen that the hydrophobicity transfer speed and the asymptotic value of the contact angle are both influenced by the relative humidity. The transfer speed and the asymptotic value of the contact angle decrease significantly, especially when the relative humidity is high. Figure S7B shows the CAs of microstructure array as a function of UV irradiation time. It was found that the CA declined a little from 168.3° to 163.2° after the first 12 h UV exposure, however showed no great change for further irradiation. Also it should be noted that the sample surface has still remained superhydrophobic with a CA of 158.9° before UV exposure (Fig. S7B) after 384 h, i.e., 16 days, of UV exposure. This indicates that the superhydrophobicity of the microstructure array possesses excellent resistance to UV light. Figure S7C shows the relationship between the water CA and SA of asprepared superhydrophobic surface and the exposure time. The CA on the surface only slightly decreased to 158.5 ± 0.9° from 163.3 ± 0.7° after exposure more than 24 days, as well as the SA is less than 10°, exhibiting the long-term stability of the resulting surface. This demonstrates that the long-term stability of the obtained superhydrophobic surface is of great importance to the practical application of superhydrophobic surface.

In order to analyze the chemical stability, the influence of pH of an aqueous solution and saline solution on the superhydrophobicity was studied for the as-prepared surface. A dry sample was placed inside a closed experimental cell, with water vapors saturated with respect to the test solutions. Figure S7D shows the evolution of the CA formed by drops of aqueous solutions of H_2_SO_4_ with pH = 1, NaOH with pH = 13 and neutral 3.5 wt.% NaCl solution for the superhydrophobic surface. From Fig. S7D, we can find that the CA is higher than 150° after 24 h of contact with H_2_SO_4_ and NaCl solutions, suggesting a good stability. However, the CA is lower than 150° after 3 h of contact with NaOH solution and finally decreased to 124° after 24 h of contact with NaOH solution. This is due to the occurrence of the chemical reaction between stearic acid and NaOH. It was investigated the pH and surface wetting properties changing before and after the reaction. Through the rate of the self-assembly reaction process carry on, the pH value decelerated and the CA increased first then decreased in Fig. S7E, F. In this case, pH change in solution reaction could affect the microstructure and hydrophobicity.

Therefore, we propose that our triphasic approach is a potentially versatile approach to directly position hierarchical combinatorial architectures on to the surfaces of pillar shaped PDMS substrates, thus producing self-assembled superhydrophobic materials without the need for multiple operational processes and complicated instrumentation.

## Conclusion

A triphasic interface-mediated strategy has been used to attach hierarchical silica biomorph microstructural arrays to the surfaces of vertical hydrophobic micropillars PDMS substrates. The factors that affect solid/liquid/gas interface coprecipitation of the local chemical field have been evaluated to identify what parameters are responsible for controlling the self-assembly reaction at the growth front, which has enabled conditions to be identified that enable the morphology, size and direction of the resultant self-assembled structures to be controlled. Triphasic interface growth behavior was found to occur via liquid-gas and solid-liquid growth processes, with overall growth rates controlled by the ambient CO_2_ concentration at the site of reaction. The artificial hierarchical structure shows remarkable super-hydrophobic property and petal effect. The super-hydrophobic property and tunable adhesion are related to the number of arms and the gap distance between each branch. SiO_2_-SrCO_3_ array exhibited ultrahigh durable superhydrophobic properties under UV irradiation, humidity and pH condition. It opens intriguing perspectives for designing artificial surfaces on the basis of complex hierarchical structure to form a super-hydrophobic surface. This one-step, mild fabrication method can be used to produce various different types of silica biomorph microstructure, thus providing a versatile method for fabricating materials with superhydrophobic surfaces that have potential applications for self-healing/self-regulation^31,32^, oil/water separation^33,34^ and cell culture/diagnostics/recognition^35,36^.

## Supplementary information

Supplementary information.

Supplementary information2.

Supplementary information3.

Supplementary information4.

Supplementary information5.
