# Supplementary material for: Tuning the Superhydrophobic Properties of Hierarchical Nano-microstructural Silica Biomorph Arrays Grown at Triphasic Interfaces
Source: Sci Rep. 2020 Mar 12;10:4596. doi: 10.1038/s41598-020-61530-0 (PMC7067859; doi:10.1038/s41598-020-61530-0)
Supplement: Supplementary file 1 — Supplementary information. [file 41598_2020_61530_MOESM1_ESM.docx]

**Supporting Information**

**Tuning the Superhydrophobic Properties of Hierarchical Nano-microstructural Silica Biomorph Arrays Grown at Triphasic Interfaces**

Xu-Fu Xiang^‡^, Pengjie Li^‡^, Bi-Feng Liu*

The Key Laboratory for Biomedical Photonics of MOE at Wuhan National Laboratory for Optoelectronics-Hubei Bioinformatics & Molecular Imaging Key Laboratory, Systems Biology Theme, Department of Biomedical Engineering, College of Life Science and Technology, Huazhong University of Science and Technology, Wuhan 430074, China

KEYWORDS: silica biomorphs, triphasic interface, architectural arrays, self-organization, superhydrophobic surfaces

Experiment and Characterization

(a) Experimental procedure and image analysis for real-time observation at different temperature and pH value. A PDMS micropillars array slide was vertically positioned in a 100 mL beaker containing 15 ml aqueous solution of BaCl_2_ (19.1 mM) and Na_2_SiO_3_ (8.2 mM) (Sigma Aldrich) with temperature control system on a heating device. The pH was measured using a Mettler Toledo FE20 FiveEasy pH meter and adjusted to designed pH value, and inserted the equipment to the solution for real time detecting the pH change. The process was followed in real time using a Keyence 3D optical microscope (VHX-200C) equipped with time-lapse photo function for in situ observation of silica biomorph growth at different triphasic interface (Movie S1, S2). Image analysis was performed with an in-house programmed MATLAB script that identified the formation of new structures and tracked their development as a function of time. This allowed us to measure the growth rates of nucleated sites from the time-lapse images that were taken using optical microscopy (Figure S2).

(b) UV-Durable superhydrophobicity. Cotton textile with a SiO_2_-SrCO_3_ microstructure array was placed under a Hayashi LA-410 light source, which emits UV light in the range of 320-400 nm. The light intensity was maintained at 25.0 mW/cm^2^ by adjusting the power output and the distance between the sample and the light source, which is about an order more intense than the UV intensity of normal sunlight. The water CA of the samples was measured with a drop-shape analysis system (Kruss DSA100) at five different points for each. All experiments were carried out at room temperature and ca. 30% humidity.

(c) Superhydrophobicity under different humidity condition. The samples’ hydrophobicity was determined by the contact angle (CA) method according to IEC TS 62073. The OCA20 optical contact-measuring device was used to measure the static contact angle of the samples. It was found that by using four droplets on one part of a sample, the measurement error can be within ±1°. So, four droplets of distilled water, each with a volume of 2 µL were allowed to fall randomly onto the surface. By using the special software of OCA20, the static contact angle was obtained 20 seconds after the droplets had fallen onto the surface in order to avoid time-dependent measurement errors caused e.g. by gravity or evaporation.

(d) Long-term stability. The stability of the superhydrophobicity of the obtained surface after exposure in air for various time intervals was also investigated. Fig. S7 C shows the relationship between the water CA and SA of asprepared superhydrophobic surface and the exposure time. The CA on the surface only slightly decreased to 158.5 ± 0.9° from 163.3 ± 0.7° after exposure more than 24 days, as well as the SA is less than 10°, exhibiting the long-term stability of the resulting surface. This demonstrates that the long-term stability of the obtained superhydrophobic surface is of great importance to practical application of superhydrophobic surface.

**BRIEFS****:** A novel bio‐inspired fabrication method has been developed that enables oriented three‐dimensional hierarchical silica biomorphs with defined nano-microstructures to be grown at triphasic interfaces which produces complex hierarchical structures with tunable superhydrophobic properties.


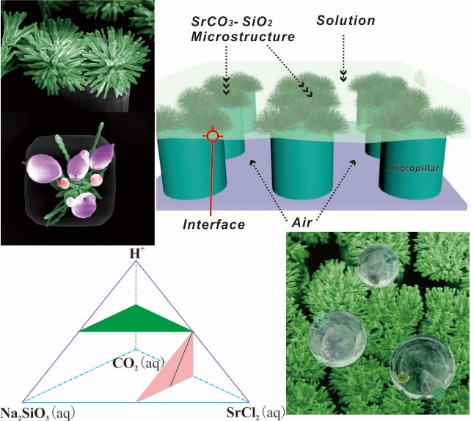


**Table S1** Range of Na_2_SiO_3_ and SrCl_2_ concentrations

|  |  | A Na_2_SiO_3_ | | | | |
| --- | --- | --- | --- | --- | --- | --- |
|  | No. | A1 (4.1 mM) | A2 (8.2 mM) | A3 (16.4 mM) | A4 (32.8 mM) | A5 (65.6 mM) |
| B  SrCl_2_ | B1 (4.6mM) | A1 B1 | A2 B1 | A3 B1 | A4 B1 | A5 B1 |
|  | B2 (9.5 mM) | A1 B2 | A2 B2 | A3 B2 | A4 B2 | A5 B2 |
|  | B3 (19.1 mM) | A1 B3 | A2 B3 | A3 B3 | A4 B3 | A5 B3 |
|  | B4 (38.2 mM) | A1 B4 | A2 B4 | A3 B4 | A4 B4 | A5 B4 |

**Table S2** Range of pH values between before and after

|  | pH 13 | pH 12.5 | pH 11.8 | pH 11.2 | pH 10 |
| --- | --- | --- | --- | --- | --- |
| A2 B1 | 1 | 2 | 3 | 4 | 5 |
| A2 B2 | 6 | 7 | 8 | 9 | 10 |
| A2 B3 | 11 | 12 | 13 | 14 | 15 |
| A2 B4 | 16 | 17 | 18 | 19 | 20 |

**Table S3** Composition and pH values of solutions used for experiments described in figure 3

| A  A5B4 pH=11.8 | B  A3B2 pH=11.8 | C  A2B3 pH=11.8 |  |  |  |  |
| --- | --- | --- | --- | --- | --- | --- |
|  | D  A3B2 pH=11.2 | E  A2B3 pH=11.8  A2B4 pH=11.8 | F  A2B3 pH=11.8  A2B4 pH=11.8 |  |  |  |
|  | | G  A3B2 pH=11.8 | H  A2B2 pH=11.8  A2B3 pH=11.8  A2B4 pH=11.8  A3B3 pH=11.8  A3B4 pH=11.8 | I  A2B4 pH=11.8  A3B4 pH=11.8 |  |  |
|  |  |  |  |  |  |  |
|  | | | J  A3B4 pH=12.5 | K  A3B4 pH=11.8  A3B4 pH=12.5 | L  A3B4 pH=11.8  A3B4 pH=12.5 | M  A3B4 pH=11.8 |


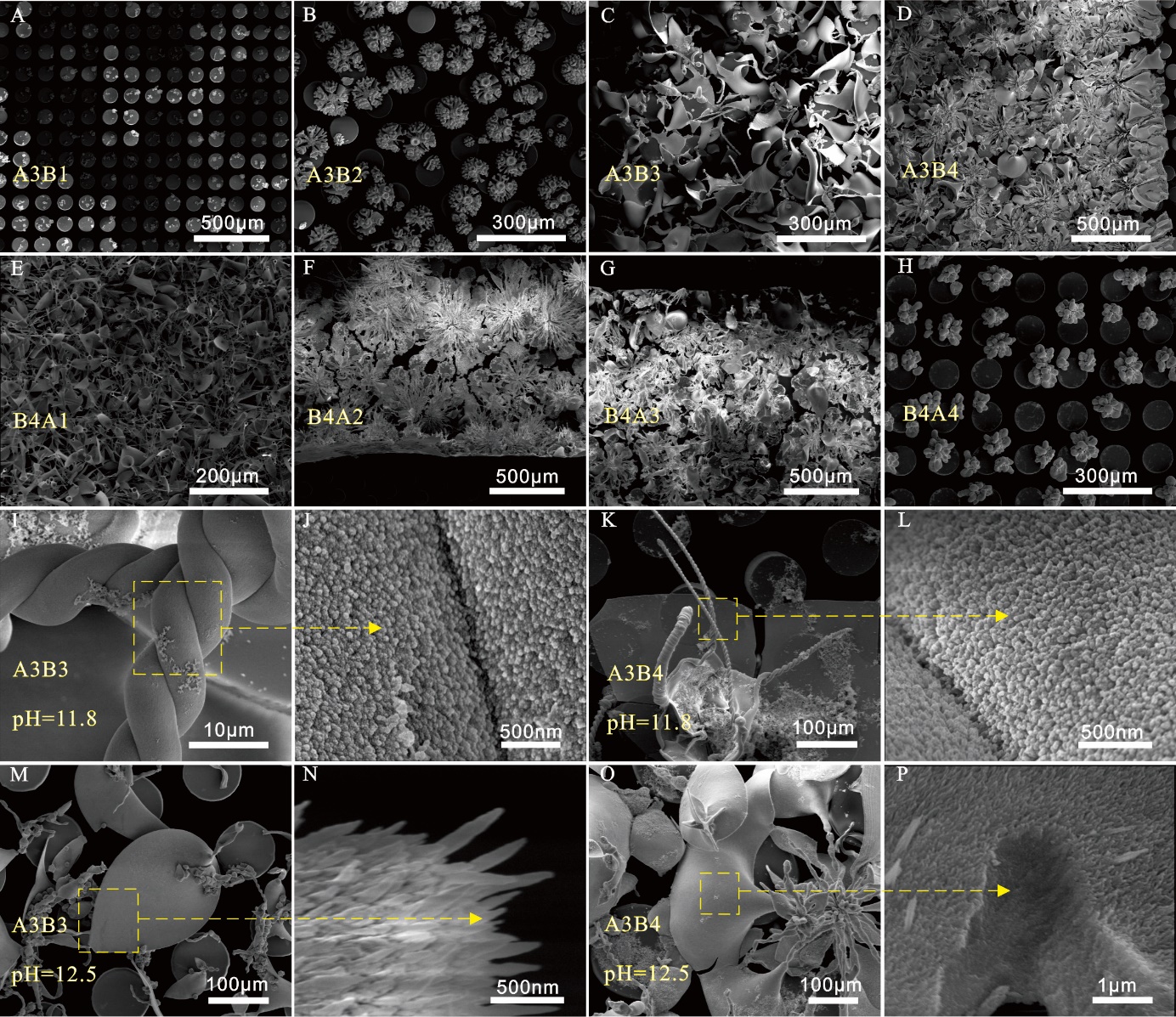


**Figure S1**. Architectural evolution of silica biomorph arrays on pillars produced using different solution concentrations (A-H) and pH values (I-P). Higher magnifications of the precipitates showed in (J, L, N, P) images, providing insight into structural details.


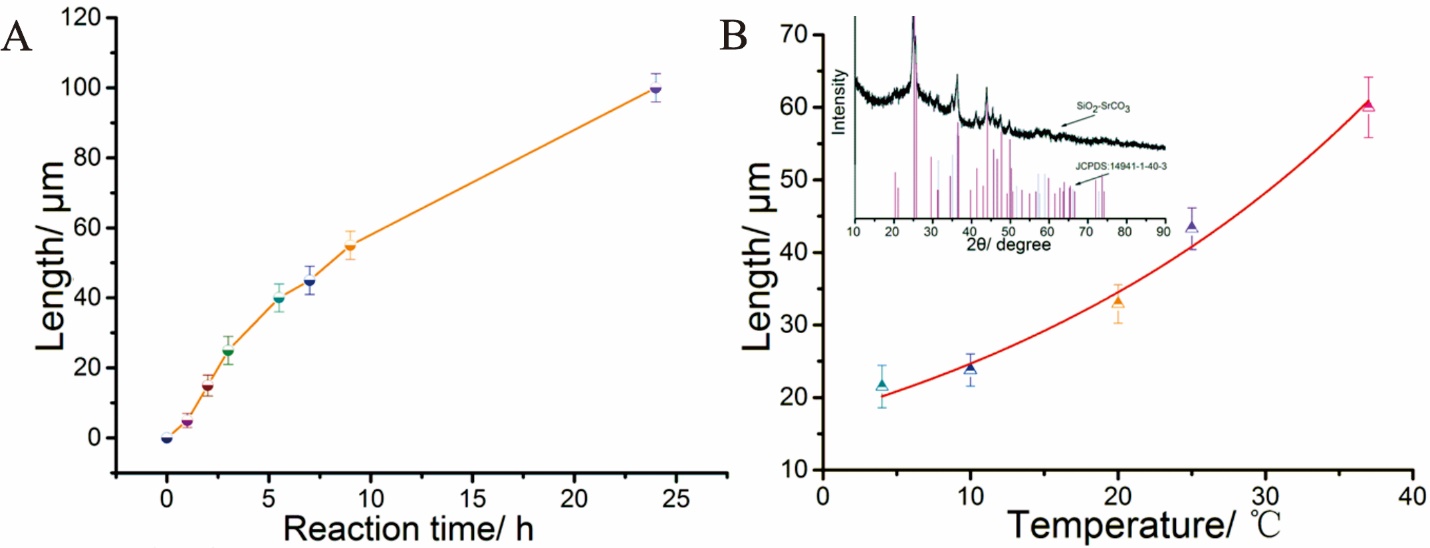


**Figure S2**. Growth details of SrCO_3_–SiO_2_ architectures deposited on hydrophobic micropillar surfaces. (A) Dependence of edge length of SrCO_3_–SiO_2_ architectures on reaction time. (B) XRD pattern of as prepared microflower arrays and dependence of the edge lengths of their SrCO_3_-SiO_2_ architectures on temperature.


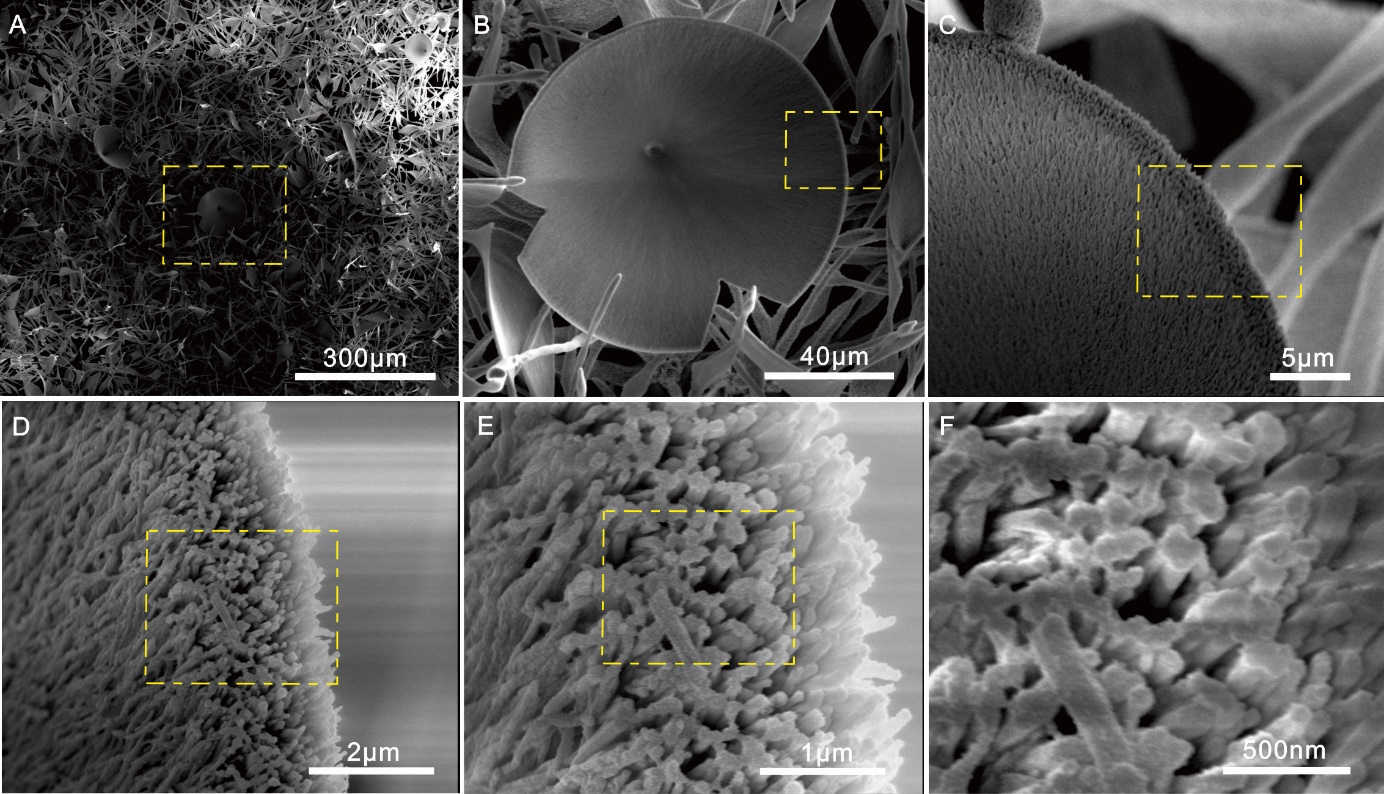


**Figure S3**. Images of the hierarchical microstructures and nanostructures of plate-like silica biomorphs, and yellow dotted line areas in SEM images represented close-up views of the amplification in proper order, revealing their microstructure.


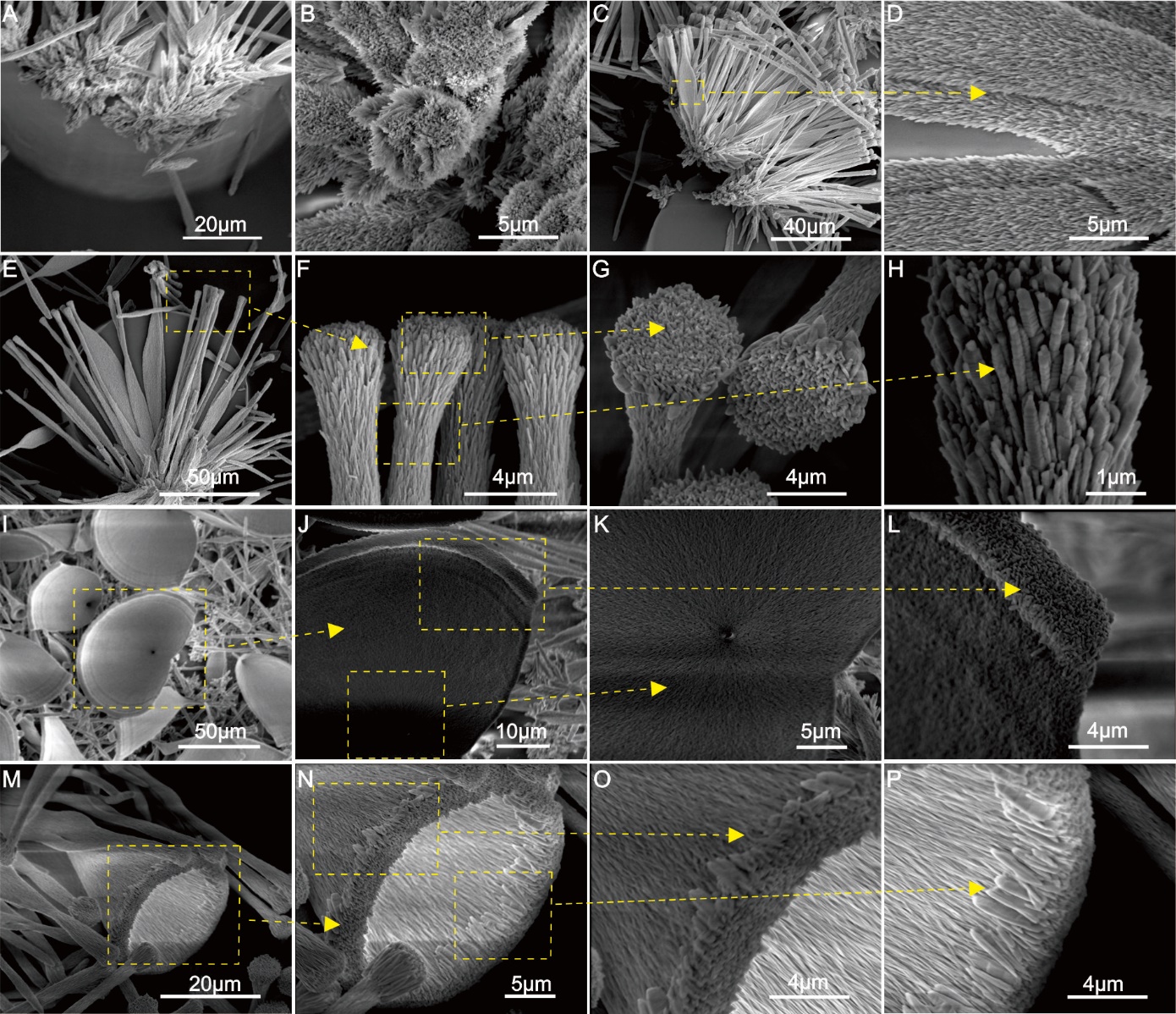


**Figure S4**. FESEM images of aggregates formed upon various shapes of carbonate crystallization, (A-H) grass-like, (I-L) flower-like and (M-P) vase-like structures of plate-like silica biomorphs. Crystals displaying elongated habits and consisting of multiple fibrous projections represent the main morphology. (C, E, I, M) Higher magnifications of the precipitates illustrated in (D, F-H, J-L, N-P) images, offering insight into structural details.


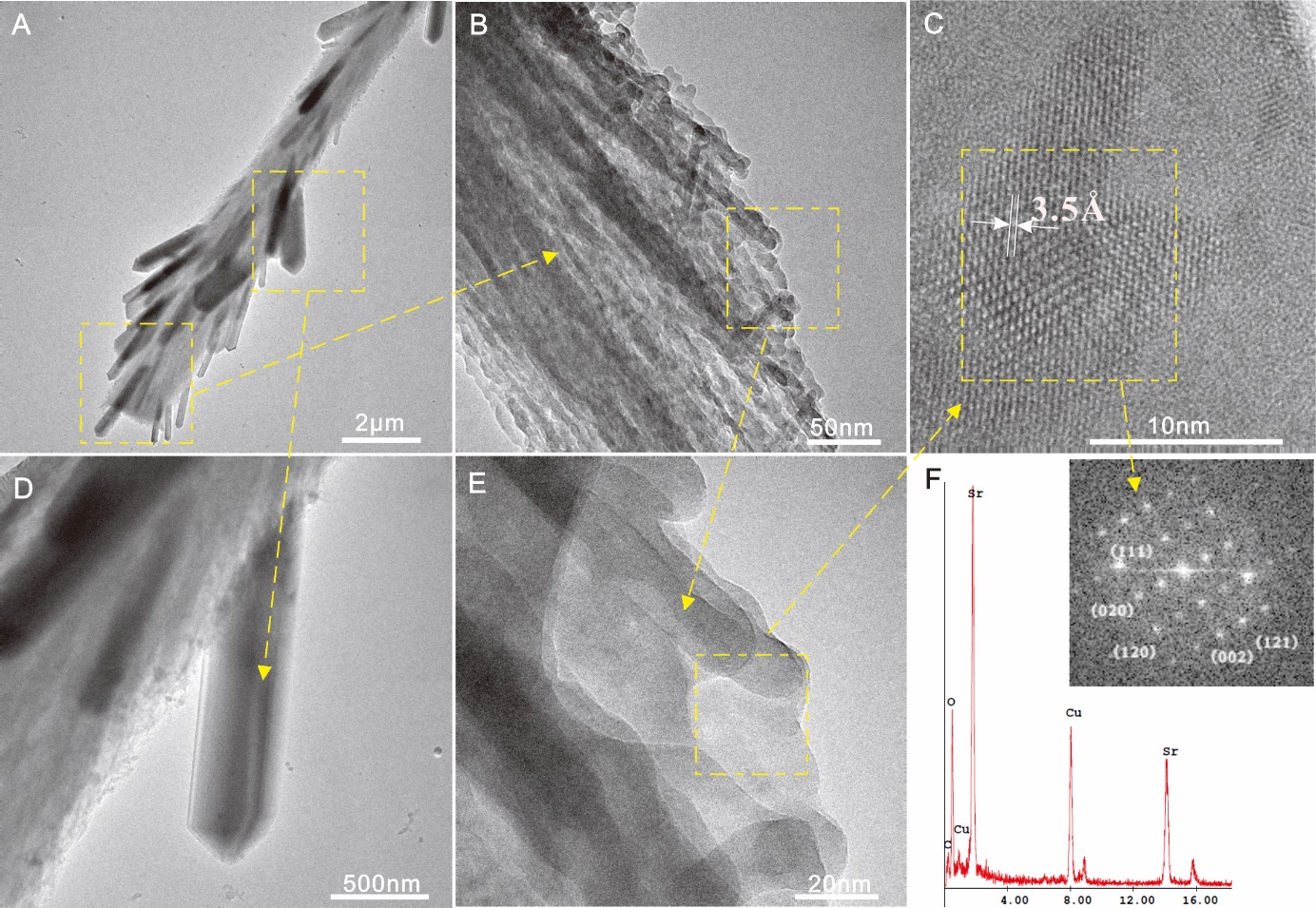


**Figure S5**. (A) Nanostructures of branch-like silica biomorphs shown a columnar 100-200 nm width SrCO_3_ nanocrystal (D), a 20-50 nm width SrCO_3_-SiO_2_ nanowire (B) and a 5-10 nm SrCO_3_ width nanoslice (E), and magnification of yellow dotted line areas showed the nanostructure detail. (C) HRTEM and (F) EDS analysis confirmed that the microstructures were comprised of SrCO_3_ crystal and composition, and (F) inserted FFT image also verified SrCO_3_ crystal structure.


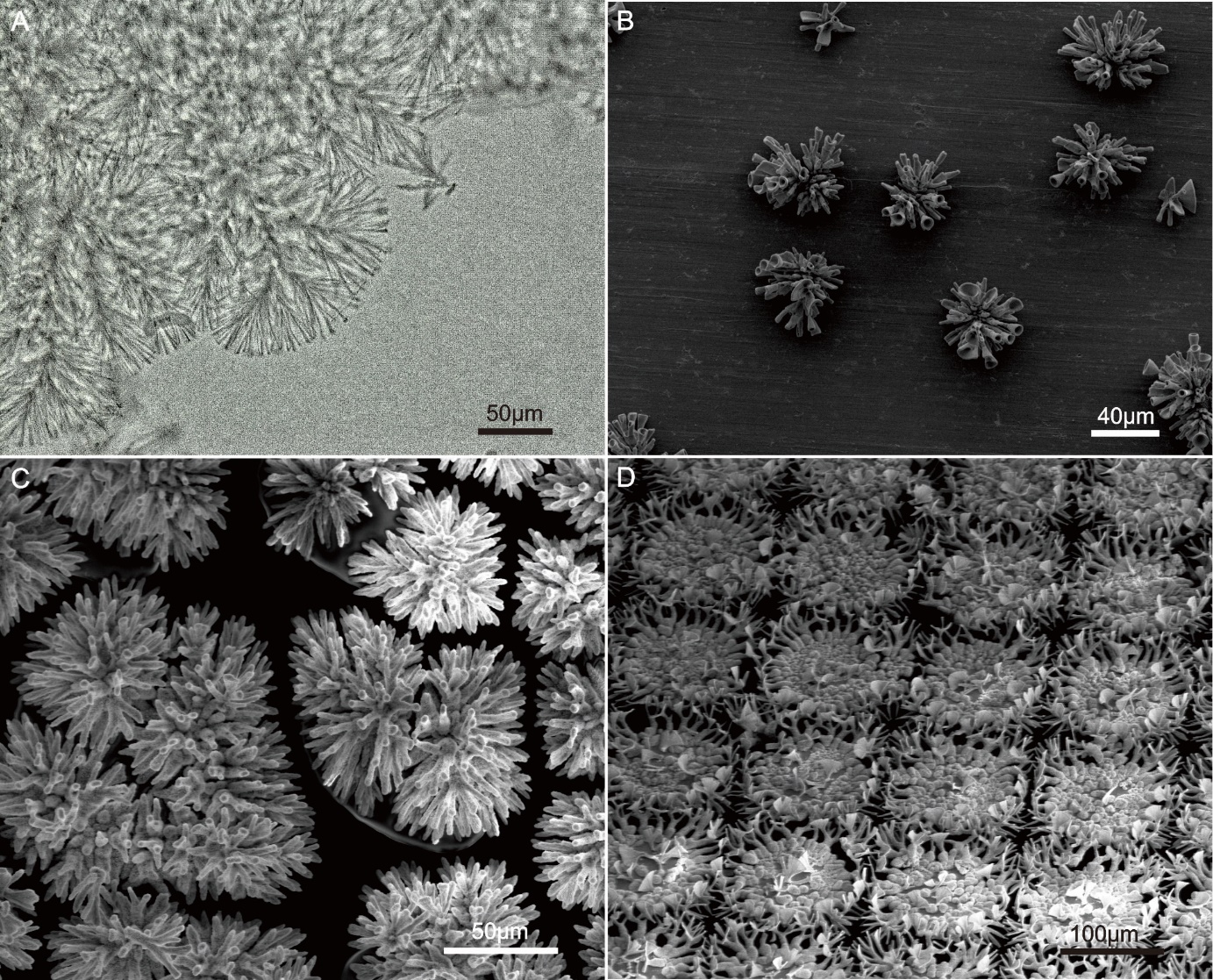


**Figure S6**. Comparison of three types of interface growth. (A) 3D-CT image of gas-liquid interface structures. (B) SEM image of solid-liquid interface formed by vertically immersion of a glass side into solution. (C-D) SEM images of gas-liquid-solid interface at a growing crystal surface.


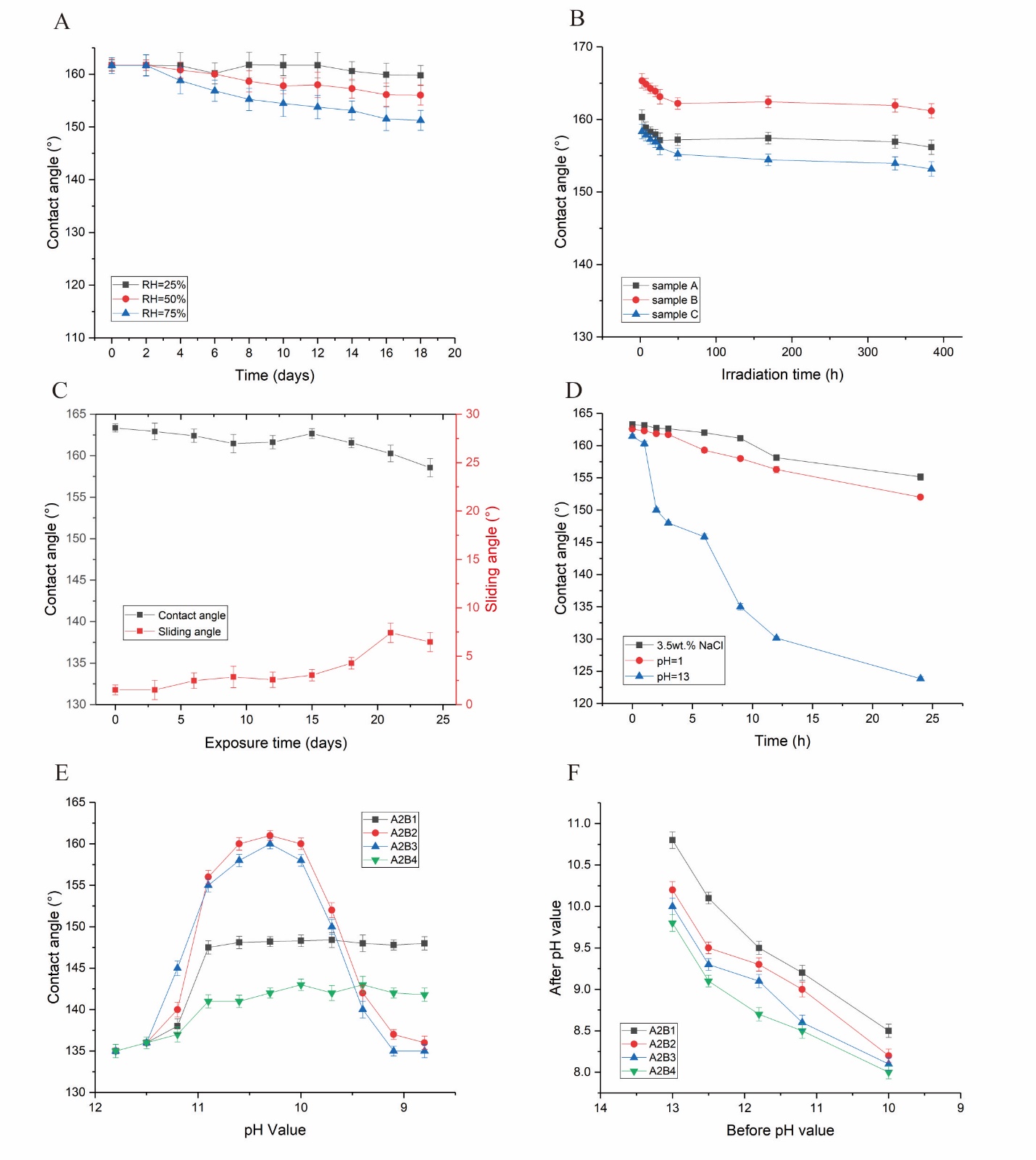


**Figure S7**. Hydrophobicity transfer characteristics of SiO_2_-SrCO_3_ array at different relative (A) air humidity, (B) UV irradiation, (C) outdoor exposure, (D, E) pH and salt solution asymptotic value of the contact angle, and (F) represented pH change before and after reaction.


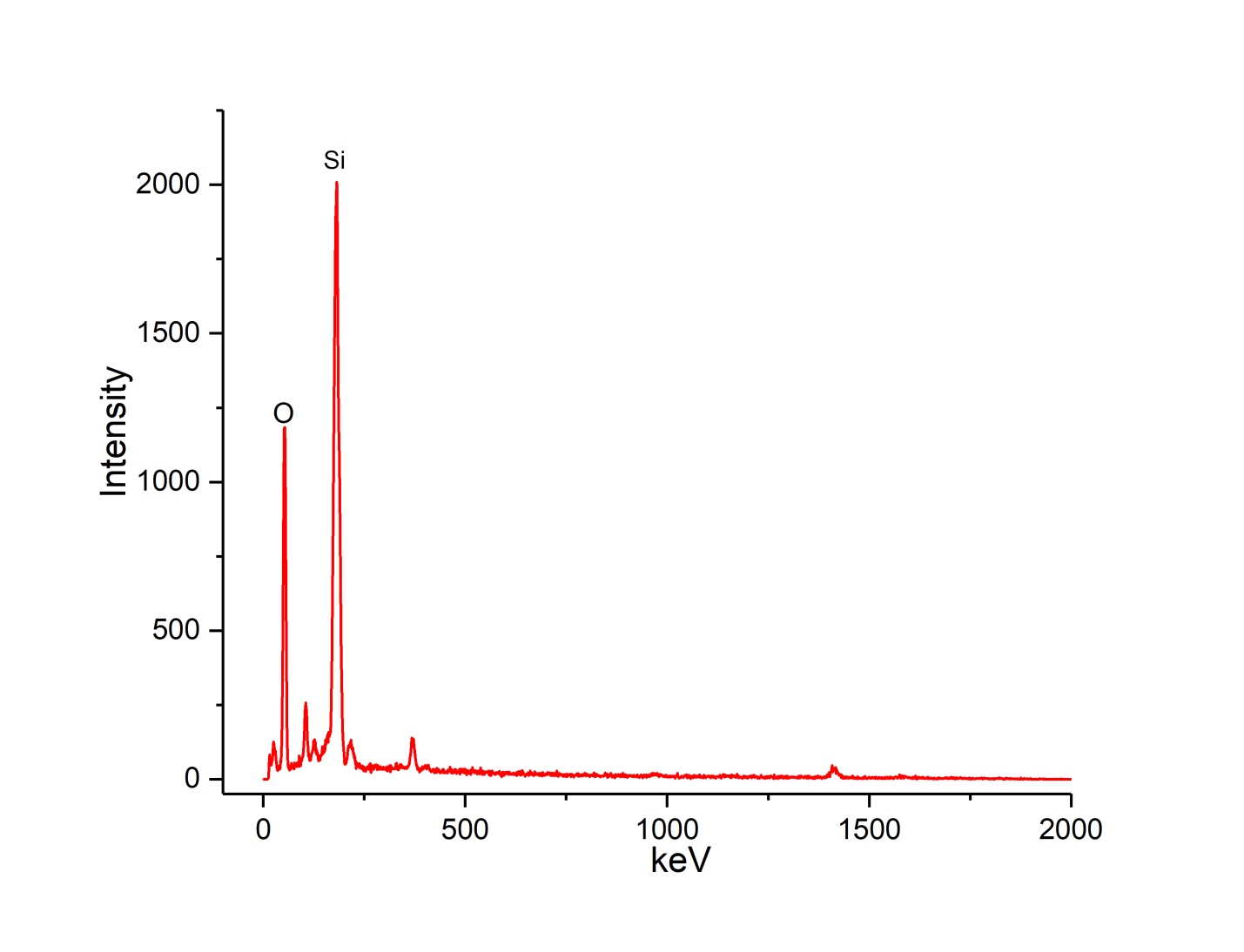


**Figure S8**. Energy dispersive spectrometric (EDS) analysis confirmed the SiO_2_ at the surface coating position of the SiO_2_- SrCO_3_ microstructure

**
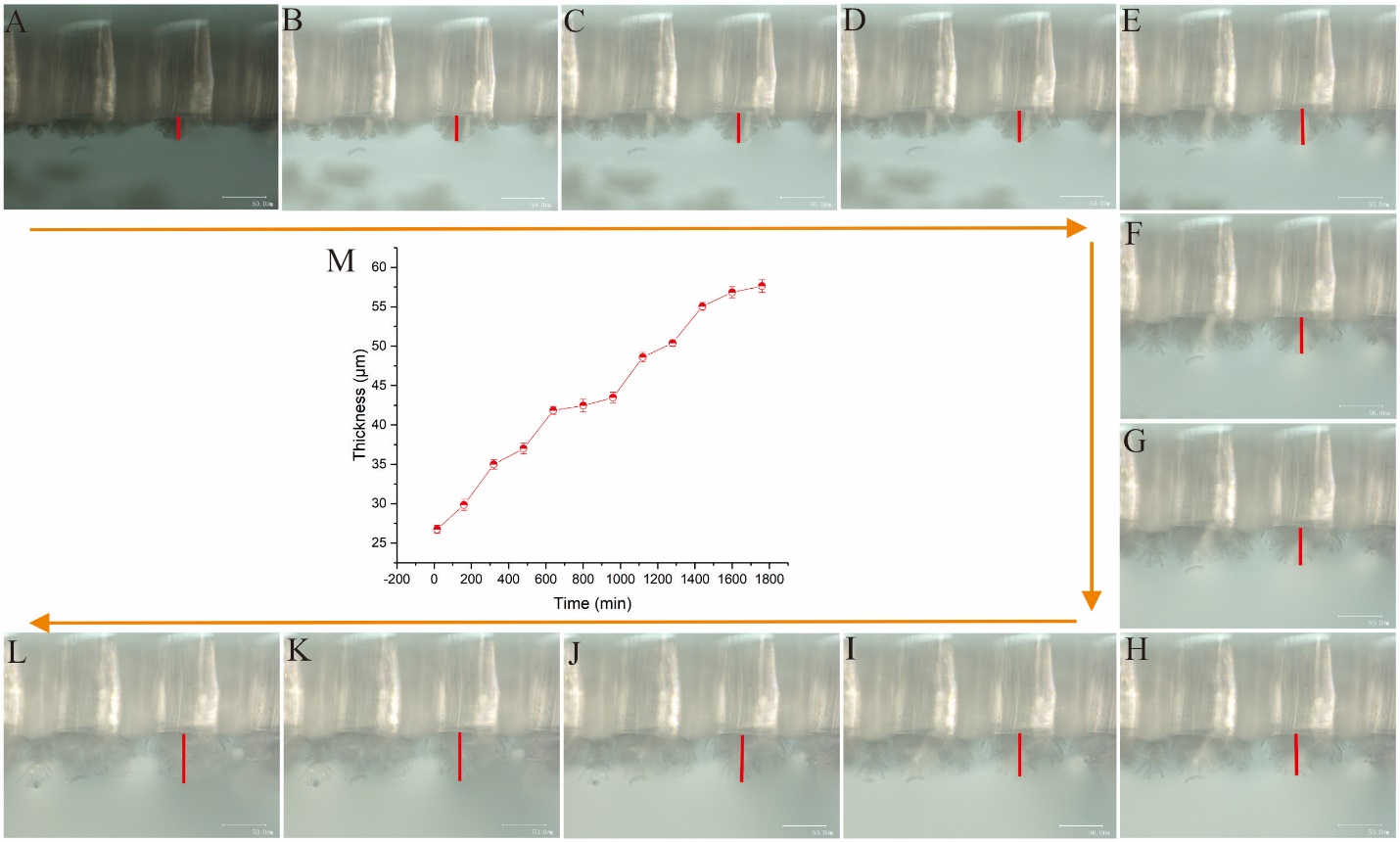
**

**Figure S9.** Thickness of SrCO_3_–SiO_2_ architectures observed by 3D microscope deposited on hydrophobic micropillar surfaces. (A-L) Top view of the microstructure growth imagine recorded per 160 minutes, (M) dependence of the edge lengths of their SrCO_3_–SiO_2_ architectures on reaction time.

Movie S1: In situ observation of silica biomorph growth at a cylinder triphasic interface.

Movie S2: In situ observation of silica biomorph growth at a rounded nose interface.

Movie S3: Captured water droplet on the surface of a hierarchical microstructure array that is then rotated through 0-180°.

Movie S4: A hierarchical microstructure array repelling a water droplet.
